# Supplementary figures and images for: 3D finite element models reveal regional fatty infiltration modulates tibialis anterior force generating capacity in FSHD
Source: PLoS One. 2025 Jul 18;20(7):e0319881. doi: 10.1371/journal.pone.0319881 (PMC12273932; doi:10.1371/journal.pone.0319881)

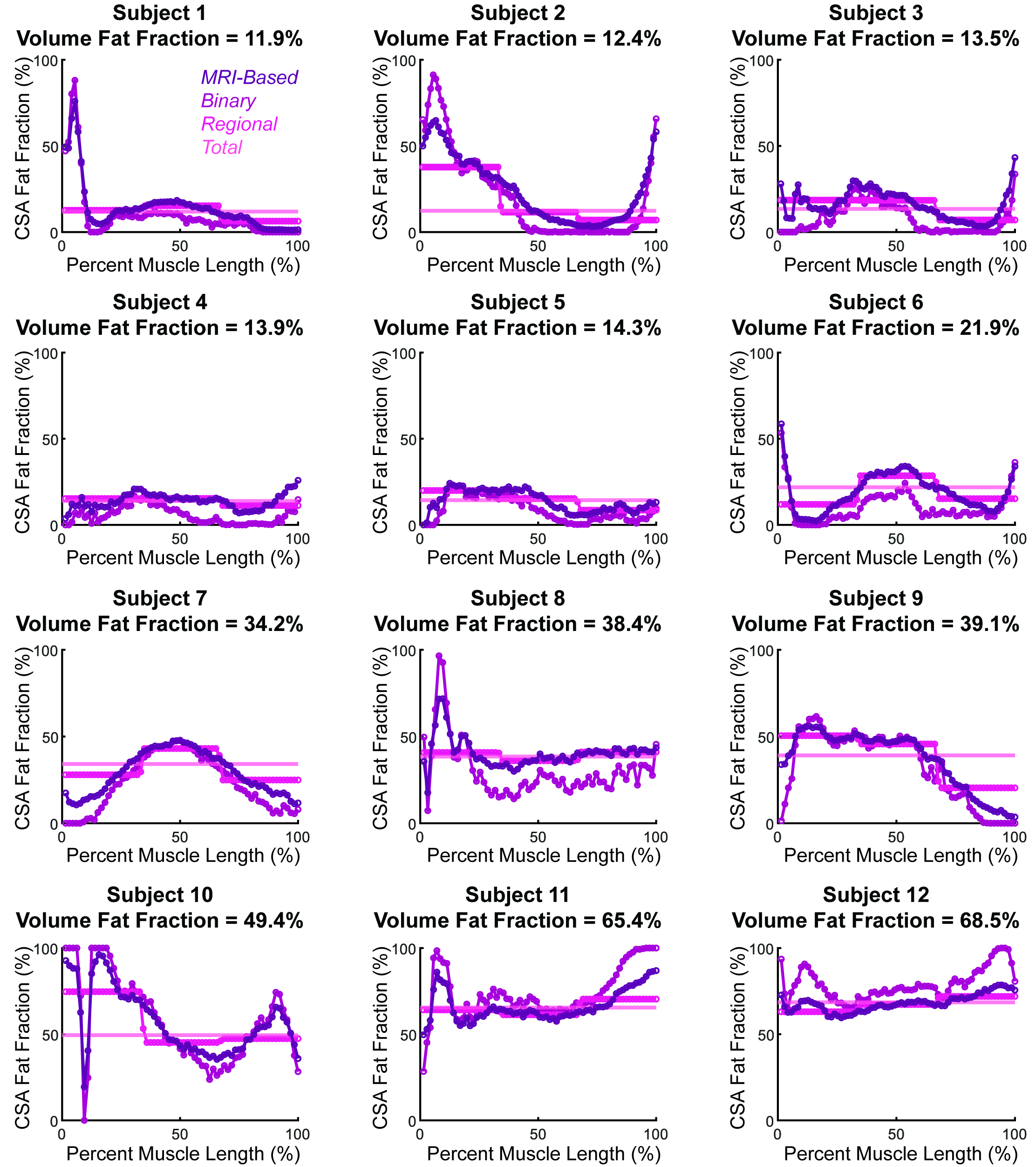

Supplement: S1 Fig — S2 Fig. (TIF) [file pone.0319881.s001.tif]

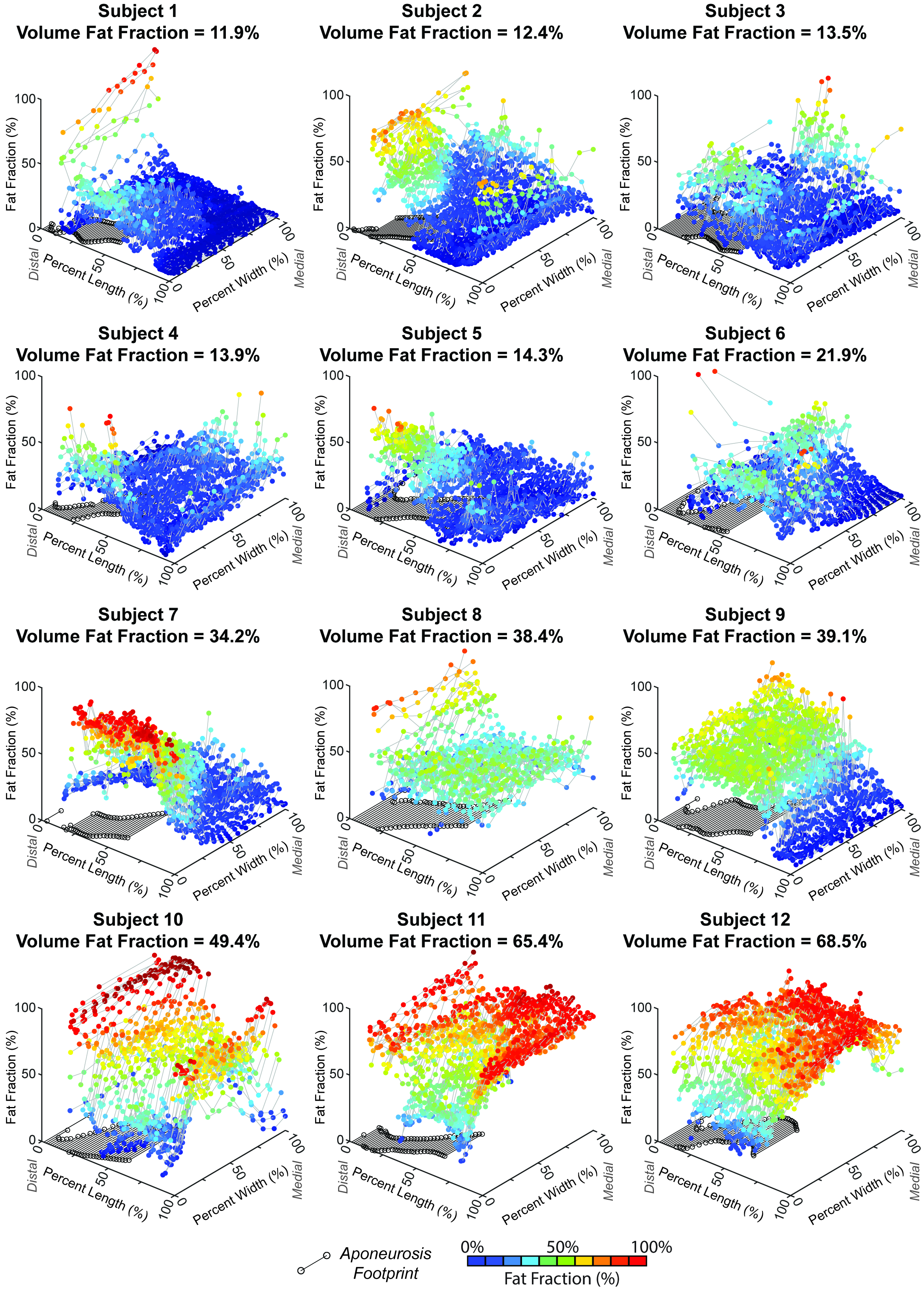

Supplement: S2 Fig — (TIF) [file pone.0319881.s002.tif]
