# Supplementary material for: 3D finite element models reveal regional fatty infiltration modulates tibialis anterior force generating capacity in FSHD
Source: PLoS One. 2025 Jul 18;20(7):e0319881. doi: 10.1371/journal.pone.0319881 (PMC12273932; doi:10.1371/journal.pone.0319881)
Supplement: Attachment — Submitted filename: Response to Reviewers.docx [file pone.0319881.s006.docx]

**Response to Reviewers: 3D finite element models reveal regional fatty infiltration modulates tibialis anterior force generating capacity in FSHD**

We graciously thank the reviewers and editors for their time and effort in reviewing our manuscript and providing edits for our accepted manuscript. Below you can find our specific responses to each comment in blue and line numbers are given from the clean draft.

*Journal Requirements:*

When submitting your revision, we need you to address these additional requirements.

1. Please ensure that your manuscript meets PLOS ONE's style requirements, including those for file naming. The PLOS ONE style templates can be found at <https://journals.plos.org/plosone/s/file?id=wjVg/PLOSOne_formatting_sample_main_body.pdf> and [https://journals.plos.org/plosone/s/file?id=ba62/PLOSOne_formatting_sample_title_authors_affiliations.pdf](https://track.editorialmanager.com/CL0/https:%2F%2Fjournals.plos.org%2Fplosone%2Fs%2Ffile%3Fid=ba62%2FPLOSOne_formatting_sample_title_authors_affiliations.pdf/1/010f0195b1050531-e19c84d9-e735-4e0f-add0-1dc941865fd4-000000/xnrUHtbgTLjdg4yXyC1RnQR9eCWVl8g8T3OHF1pqCSQ=203)

Response: We have checked and fixed any non-compliance.

2. Please note that PLOS ONE has specific guidelines on code sharing for submissions in which author-generated code underpins the findings in the manuscript. In these cases, we expect all author-generated code to be made available without restrictions upon publication of the work. Please review our guidelines at <https://journals.plos.org/plosone/s/materials-and-software-sharing#loc-sharing-code> and ensure that your code is shared in a way that follows best practice and facilitates reproducibility and reuse.

Response: All code files for model and results generation are available in the Figshare repository in the Data Availability section - <https://figshare.com/projects/3D_finite_element_models_reveal_regional_fatty_infiltration_modulates_tibialis_anterior_force_generating_capacity_in_FSHD/236591>.

3. Thank you for stating the following in the Competing Interests section:
   “The authors have read the journal's policy and the authors of this manuscript have the following competing interests: Silvia Blemker is co-founder and employee of Springbok Analytics and owns stock in the company.”

   Please confirm that this does not alter your adherence to all PLOS ONE policies on sharing data and materials, by including the following statement: "This does not alter our adherence to PLOS ONE policies on sharing data and materials.” (as detailed online in our guide for authors <http://journals.plos.org/plosone/s/competing-interests>). If there are restrictions on sharing of data and/or materials, please state these. Please note that we cannot proceed with consideration of your article until this information has been declared.

   Please include your updated Competing Interests statement in your cover letter; we will change the online submission form on your behalf.

Response: We have added the sentence “This does not alter our adherence to PLOS ONE policies on sharing data and materials.” Additionally, we have updated our competing interests statement in the cover letter.

4. In the online submission form, you indicated that “Data-sharing requests may be sent to Dr. Seth Friedman.”

All PLOS journals now require all data underlying the findings described in their manuscript to be freely available to other researchers, either 1. In a public repository, 2. Within the manuscript itself, or 3. Uploaded as supplementary information.
This policy applies to all data except where public deposition would breach compliance with the protocol approved by your research ethics board. If your data cannot be made publicly available for ethical or legal reasons (e.g., public availability would compromise patient privacy), please explain your reasons on resubmission and your exemption request will be escalated for approval.

Response: We have uploaded all segmentations, voxel-based fat maps, and contour points necessary to generate the models and results in the manuscript to the figshare site. Full images will be publicly available in a larger future public repository resulting from the NIH Wellstone project that has just completed its second of five years. Dr. Seth Friedman is the manager of the repository and thus to access the data through collaboration, data-sharing requests must be made to him until the repository is publicly available. All segmentations, voxel fat fraction maps, data and model files are available (figshare) for replication of the results presented in the manuscript.

5. Please review your reference list to ensure that it is complete and correct. If you have cited papers that have been retracted, please include the rationale for doing so in the manuscript text, or remove these references and replace them with relevant current references. Any changes to the reference list should be mentioned in the rebuttal letter that accompanies your revised manuscript. If you need to cite a retracted article, indicate the article’s retracted status in the References list and also include a citation and full reference for the retraction notice.

Response: We have checked the references and no changes were necessary.

*Reviewer's Responses to Questions*

**Reviewer #1:** The article, “3D finite element models reveal regional fatty infiltration modulates tibialis anterior force generating capacity in FSHD”, is a hypothesis generating, technical article that aims to 1) develop a pipeline for creating subject-specific models of the TA that include fat infiltration patterns measured from MRI and predict force generation, 2) compare models created using this pipeline with clinical measures of muscle strength, and 3) use the models to investigate the impact of regional fat distribution on muscle force generation. The authors developed 3D finite element models (FEMs) to create subject-specific models incorporating fat infiltration patterns derived from MRI data. 12 subject specific models were created. They found that the model-predicted forces correlated cross-sectionally with clinical measures of strength (MMT>QMT) and that that fat amount accounts for 48% and muscle volume accounts for 74% of the variation in force. The creation of the 8 pseudo-maps enabled systematic variation of fat location and amount while holding all other parameters constant allowing for assessment of regional fat distributions on muscle force generation. They simulated different fat distributions (proximal to distal, anterior to posterior and medial to lateral) and found that fat location modulates force generation (mostly proximal to distal) with fat in the middle region having the greatest impact on reducing force. The authors also used ultrasound for 2D to 3D comparison to evaluate cross sectional area comparisons. Pearson correlation was used to compare model-generated force prediction with clinical strength tests (MMT and QMT) and sensitivity analysis was performed by varying fat shear modulus to assess its impact on force predictions.

The authors concluded that the FEM shows a more linear relationship between fat fraction and normalized force than previously published. They suggest that muscle atrophy and fat infiltration happen in parallel and therefore contribute to the non-linearity in real world data and that by controlling for volumetric and muscle shape effects, that fat infiltration has a direct impact on muscle force generation. While there is a direct relationship with fat fraction and force, it seems that there is a stronger relationship with lean muscle volume. The authors acknowledge this finding and suggest calculating both lean muscle volume in addition to fat fraction.

It is interesting that there was stronger agreement with MMT, a gross measure of muscle strength, rather than QMT which is thought to be more precise. They explain that this outcome was expected as QMT measures force for a joint level dorsiflexor torque, along with additional normalizations applied that prevent it from being converted into an estimated muscle force for a more direct comparison.

Questions for the authors:

1. In line 91, the authors mention that participants were chosen based on several different clinical features. Is it possible to include genetic characterization, e.g. number of repeats? If number of repeats are available, is there any correlation?

Response: Indeed, we selected participants from a future repository primarily based on the functional test scores provided, QMT and MMT. We have included the simplified allele length for additional genetic characterization in Table 1. Interestingly we found no significant correlation between allele length and model force or QMT. The only functional quantity with a significant, although weak, correlation to allele length was MMT (R^2^ = 0.38). We added a sentence on the no significant correlations with simplified allele length and model force in line 257-258.

2. The TA was segmented with the used of AI and manual vetting. Would it be possible to automate the entire process?

Response: While full automation is possible, it is more challenging in this case given the complex morphology of fatty-infiltrated muscles. Therefore, we believe that the addition of manual vetting to ensure the accuracy of the results is warranted.

3. Lines 225 – The authors describe how they predicted force. Does this take into account age?

Response: We do not account for age in the force prediction as the same constitutive material is used for the muscle in all the models. Current efforts in modeling aging muscles in healthy people use several methods to account for the change in muscle composition with age, such as adding passive scaling terms (Liang et al., 2024), reducing activation (Giantesio & Musesti, 2017), or modifying the material properties of the constitutive model (Arya & Singh, 2022). Since our models are directly derived from images and incorporate the specific composition of each muscle, they inherently incorporate any effect of age on muscle composition.

4. In 230, the authors describe ultrasound measurements. I am not sure about the intent of the authors including the data from US as the relationship between the 2D US and 3D MRI. This data again seems to indicate that the better measure associated with force is muscle volume rather than fat fraction. The results from evaluation of the model as applied to US shows that CSA at 60% the length of the TA was strongly related to muscle and force. Are you implying that this measurement at 60% the length of the TA is a good assessment of the entire muscle volume? Could it be that US is a cheaper, easier method to assess association of fat replacement with muscle strength and potentially predict disease progression?

Response: There has been increasing interest in using ultrasound to evaluate muscles in patients with NMDs - including our own work in Duchenne muscular dystrophy and spinal muscular atrophy (McCrady et al., 2024). Therefore, we included an ultrasound-like assessment as an exploratory result, since this is a unique opportunity to assess 3D data in a 2D manner, therefore providing support for ultrasound-based muscle assessments. We agree that MRI quantification of muscle volume is the gold standard, however additional monitoring at a lower cost may be feasible with ultrasound. It is well established that muscle cross-sectional area correlates to muscle volume in healthy people, therefore it can serve as a surrogate measure for muscle volume. Future work collecting ultrasound and MRI of the TA (and additional muscles of interest) would be essential to build on these results for transition to a clinical assessment.

5. Line 237 Why did variation of shear modulus in 3 subjects lead to no significant change? Is it because of the low fat fraction or because of lean muscle volume?

Response: This analysis is modulating the fat properties since intramuscular fat shear modulus is not characterized in the literature, and other intramuscular fat modeling studies use a range of fat moduli for constitutive modeling. The results show that there is no sensitivity to the fat modulus within the physiological range for the force output, allowing us to have confidence in the 4.19 kPa used for the fat constitutive model. If we allow the fat shear modulus to be outside the physiological range at 500 kPa, we the force output decreases, showing sensitivity (see new Fig 5). These models have a range of volumetric fat fractions and lean muscle volumes, which is reflected in the total force output for each model. We have modified the caption and added language on this in line 248.

6. Please clarify if you will be able to apply this model to all muscles of the body or if additional models will need to be generated. You mention on line 363 and 389 that specific muscle geometry and fat infiltration patterns will be important for all analyses. Can you provide an overview of which muscles would share similar features?

Response: Additional models of specific muscles of interest need to be generated to assess the response across various architectures. This same model generation pipeline can be applied to other muscles across the body, allowing for muscle specific models to be generated using the MRI segmentations and available fiber characterizations in the literature. These findings may be specific to the TA due to its complex architecture, therefore follow on work should investigate modeling additional muscle of interest in FSHD such as the trapezius and shoulder girdle muscles. Muscles that share similar architectural features as the TA are bipennate muscles with a central aponeurosis (e.g. rectus femoris, rectus abdominals).

7. How will inclusion of lean muscle volume potentially change the model? It seems that there is a higher correlation of force with lean muscle volume than with muscle fat fraction. They suggest that muscle atrophy and fat infiltration happen in parallel and therefore contribute to the non-linearity in real world data and that by controlling for volumetric and muscle shape effects, that fat infiltration has a direct impact on muscle force generation.

Response: The lean muscle volume is calculated from the finite element model; therefore, it is already accounted for in the finite element model. For our regression models, we presented univariate models, however we have generated an additional multivariate model incorporating volumetric fat fraction and LMV to estimate muscle force (adjusted R^2^ = 0.90). This model was not significantly improved compared to the univariate model of LMV alone. This model was significantly improved in force estimation compared to fat fraction alone, suggesting that LMV is a better indicator of function than fat fraction alone.

8. While there is moderate to excellent cross sectional correlation of fat fraction/location of fat fraction and lean muscle volume respectively, what parameters/clinical evaluations do you think will be important for assessment of longitudinal correlations?

Response: Thank you for this question, in fact we are already moving towards longitudinal modeling studies. When considering longitudinal correlations, we posit the inclusion of fat fraction and LMV are essential for characterizing a functional response. Based on the results of this work, fat infiltration location is likely an important factor in explaining some of the clinical variation in force generation as it likely relates to the architecture and functional compartments of the muscle. Future work is necessary to link the mechanical perturbations (i.e. alterations in strain patterns) due to fat infiltration with fat propagation through longitudinal and multiscale models.

**Reviewer #2:** This paper reports MRI-based 3D finite element models to show the relationship between fatty infiltration and force generation in TA muscles in FSHD. This study aimed to develop a pipeline for creating subject-specific models of the TA, to compare models created using this pipeline with clinical measures of muscle strength, and to use the models to investigate the impact of regional fat distribution on muscle force generation by twelve subject-specific modeling. This work is very interesting and logical for interpreting how fat replacement contributes to the decline in force generation. The introduction and discussion are well-written. However, there are difficulties to read.

1. At first, why was TA used for this study, not the facial, scapula, and humerus muscles?

Response: When FSHD was first characterized, it was initially thought to primarily impact muscles in the face, shoulder, and upper arm – hence the name. However, recent work has highlighted the pattern of involvement is not limited to the upper extremity and may be heterogenous in the patient population (Rijken et al., 2014). We used the TA muscle due to growing interest in the pattern of early TA involvement in FSHD. Additionally, we collected lower extremity MRIs, limiting our assessment to lower extremity muscles. It is certainly possible to apply this framework to other highly affected muscles in FSHD such as the facial, shoulder, and upper extremity muscles with MRIs of those areas. Indeed, recent work from Springbok has developed robust segmentations of the whole-body thereby making it possible to apply this modeling pipeline to other key muscles in FSHD. Per the reviewer’s comment, we have added a sentence justifying the focus on the TA muscle on line 48-49 and 95-97.

2. Figure legends are not enough to explain the contents of Figures.
   In Fig2, Fig3, Fig4, Fig5, Fig6, Fig8, only titles were provided. I could not get what is shown in Figure 8. Need the explanation for all Figs.

Response: We apologize that the captions were not sufficient for understanding the figures. We have added more explanation to the captions of the figures where there were only titles. Additionally, we realized the color scale bar for Fig 8 was not correct as it reflects the increasing fat fraction within the participant group, not the actual value of the fat fraction. We have edited this Figure for clarity. We have provided additional explanations for each of the figures (new order with Fig 2 moved to Fig 7) below:

1. This figure shows the model development pipeline in a graphical format
2. This figure is a graphical representation of the definition of the regions of fat infiltration in the pseudo mapping analysis
3. This figure shows the beta distribution probability density functions used to apply fat fractions to each element in the pseudo maps
4. This figure is a graphical representation of the boundary conditions of the finite element model based on the boney attachments of the TA and its tendons
5. This figure shows the results of a sensitivity analysis on the force output for various fat stiffnesses. This plot shows there was no sensitivity in force generation in response to increasing fat stiffness within the physiological range, however outside the expected range there is model sensitivity to the fat constitutive model parameters.
6. This figure shows the correlation of the model force to the QMT and MMT clinical function scores
7. This figure shows the variation in anatomical fat infiltration patterns we modeled
8. This figure shows the force for all the variations in the anatomical fat infiltration maps compared to the healthy muscle, demonstrating the non-uniform force loss across a range of fat fractions and infiltration patterns
9. This figure shows the percent force loss compared to the fat fraction in the first column. This analysis normalized the force to account for the volume effects of force loss, showing the direct effect of fat on force loss. The second column shows the regressions of fat fraction to force. The final column shows the lean muscle volume to force regressions.
10. This figure shows the exploratory analysis of simulating an axial ultrasound scan and its potential utility in clinical muscle assessments in FSHD
11. This figure shows the force loss compared to fat fraction for all 8 pseudo maps, highlighting the region infiltrated modulates the force loss.
3. Figure 2 is interesting and should be moved to the Result section.

Response: Thank you, we also appreciate the visual representation of the fat infiltration patterns. We have moved this figure to the Results section. It is now Fig 7.

4. The authors said a more linear relationship between fat fraction and normalized force as shown in Fig 9. On the other hand, they concluded that the location of fat infiltration in the TA muscle volume modulates force-generating capacity, with fat infiltration in the middle compartment being especially detrimental, as in Fig11. However, fat distribution in TA is heterogeneous from patient to patient in Fig2. If the regional fat volume might have more effects on force generation, it should result in a non-linear relationship among the patients. Additionally, Fig10 shows that higher R2 was given by use of volume fat fraction, not midbelly fat fraction. Please explain that point.

Response: Thank you for the observations. In Fig 9, we see in the first column a linear relationship between normalized force and volumetric fat fraction. This normalization uncouples the fat and volume effects on force, allowing us to see there is a direct impact of fat amount on force loss. In the middle column, we are looking at the force output for the patients. Particularly, in Fig 9B, we see the MRI-based representation of the fat infiltration with force versus fat fraction. This plot shows the non-linearity that appears in typical clinical data comparing QMT and fat fraction. This is likely due to the heterogenous infiltration patterns in the muscles modeled. In Fig 10, which was an exploration of a 2D simulated measurement to test the feasibility of an ultrasound-based assessment shows a slightly higher R2 when using MRI volume based fat fraction compared to the midbelly fat fraction of one cross section, however the same non-linearity is present in both plots and the R2 values are similar, suggesting the 2D assessment is similar to the 3D assessment. In Fig 11, the results of the simulated fat infiltration patterns are shown to systematically assess location impacts of fat infiltration. Here we see that fat infiltration of the middle region of the shows a steep decline in force as the middle region becomes increasingly fatty, compared to other infiltration patterns. This is not necessarily a clinical pattern; however, it suggests that as various regions are infiltrated, we will get a heterogenous force versus fat fraction relationship due to the specific regions impacted.

5. Similarly, the relationship between Force and Lean muscle volume gave a high correlation coefficient R^2^>0.9. So total fat volume has more impact on force generation.

Response: Indeed, LMV showed high correlations with force. This is because muscle volume is the primary driver of the amount of force generated and thus, we would expect a direct relationship when calculating the amount of muscle versus force. Fat volume has a large impact on force generation; however, it is characterizing the non-contractile volume in the muscle, and that may change independent of the LMV. Therefore, fat volume may not be a strong indicator of force generation since we posit that volume loss and fat infiltration happen in parallel and may change independent of each other. This aligns with our conclusion that fat fraction alone is not sufficient in assessing function of the TA, and that we must account for the contractile muscle volume present in the muscle.

6. Please clarify that Fig10F is the same as Fig9B.

Response: Thank you, we have added this to the caption of Figs 9 and 10.

**References:**

Arya, K., & Singh, A. (2022). Active behavior of skeletal muscle and modeling sarcopenia with a mixture active strain approach based constitutive model. *International Journal of Solids and Structures*, *248*, 111669. https://doi.org/10.1016/J.IJSOLSTR.2022.111669

Giantesio, G., & Musesti, A. (2017). A Continuum Model of Skeletal Muscle Tissue with Loss of Activation. *Lecture Notes in Computational Science and Engineering*, *122*, 139–159. https://doi.org/10.1007/978-3-319-73371-5_7

Liang, C., Jiang, F., Kawaguchi, D., & Chen, X. (2024). A Biomechanical Simulation of Forearm Flexion Using the Finite Element Approach. *Bioengineering*, *11*(1), 23. https://doi.org/10.3390/BIOENGINEERING11010023/S1

McCrady, A. N., Masterson, C. D., Barnes, L. E., Scharf, R. J., & Blemker, S. S. (2024). Development of an ultrasound-based metric of muscle functional capacity for use in patients with neuromuscular disease. *Muscle & Nerve*. https://doi.org/10.1002/MUS.28263

Rijken, N. H. M., van der Kooi, E. L., Hendriks, J. C. M., van Asseldonk, R. J. G. P., Padberg, G. W., Geurts, A. C. H., & van Engelen, B. G. M. (2014). Skeletal muscle imaging in facioscapulohumeral muscular dystrophy, pattern and asymmetry of individual muscle involvement. *Neuromuscular Disorders*, *24*(12), 1087–1096. https://doi.org/10.1016/J.NMD.2014.05.012
